# The hatching of consciousness

**DOI:** 10.1007/s40656-021-00472-w

**Published:** 2021-11-22

**Authors:** Jonathan Birch

**Affiliations:** Centre for Philosophy of Natural and Social Science, London School of Economics and Political Science, Houghton Street, London, WC2A 2AE UK

**Keywords:** Evolution, Consciousness, Subjective experience, Phenomenal consciousness, Sentience, Emotion

## Abstract

Peter Godfrey-Smith’s *Metazoa* and Joseph LeDoux’s *The Deep History of Ourselves* present radically different big pictures regarding the nature, evolution and distribution of consciousness in animals. In this essay review, I discuss the motivations behind these big pictures and try to steer a course between them.

Books reviewed

Godfrey-Smith, Peter. (2020). *Metazoa: Animal minds and the birth of consciousness*. London: William Collins.

LeDoux, Joseph. (2019). *The deep history of ourselves: The four-billion-year story of how we got conscious brains*. New York: Viking Press.

Reading these books together induces a strange feeling. In some ways, they are so similar that, had the publishers swapped the titles while the books were in press, no one would have noticed. In other ways, they are so different that it is hard to believe they are about the same thing at all. Some quotations will give a sense of what I mean:

Once we accept that there is probably some form of experience in hermit crabs, octopuses, and so on, there’s a need for a broad view - a broad account of what they have, and we have, that makes us all experiencing beings. … Human experience is a mixture of old and new. (Godfrey-Smith, 2020, p. 269)

The picture of consciousness I’ve painted so far is extremely anthropocentric—human centered—as it should be. Human consciousness depends on unparalleled cognitive processes that are entwined with language and culture, and is enabled by circuits with unique properties. The evolutionary past of human consciousness … is, in my view, shallow rather than deep. (LeDoux, 2019, p. 316)

Sentience is not absolutely everywhere, even within life. But there is a lot of it, from sea angels (perhaps) to sea dragons (for sure). The world is fuller, more replete with experience, than many people have countenanced. (Godfrey-Smith, 2020, p. 279)

For my part, I assume brains were nonconscious long before they were conscious. So my default position is that behaviour is controlled nonconsciously until proven otherwise. … [T]he human brain is the only physical system that unequivocally possesses consciousness. (LeDoux, 2019, p. 328)

You might think they must mean something different by experience/consciousness, but they insist they mean the same thing. Both are referring to subjective experience, alias “phenomenal consciousness”, or consciousness in the Nagel (1974) sense: what it’s like (or feels like) to be you (Godfrey-Smith, 2020, pp. 13–16; LeDoux, 2019, p. 271).

I could string together pages and pages of quotations like those above, creating an imaginary dialogue between wildly opposing views. That would be fun, but would not do much to move the debate forward. What I want to do instead is give each book some time in the spotlight, so as to give a sense of *why* the authors are led to such different end-points—and then see if I can at least partially reconcile them.

## Minimal subjects

*Metazoa* is a loose sequel to *Other Minds* (Godfrey-Smith, 2016)—loose enough that it can easily be read as a standalone book. Indeed, a closer relative than *Other Minds* is Godfrey-Smith’s (2019) article “Evolving across the explanatory gap”. Although written accessibly and for a wide audience, *Metazoa* is still a serious work of philosophy, and anyone with an interest in the philosophy of animal minds will find it stimulating and provocative. Through discussions of protists, sponges, cnidarians (jellyfish, sea anemones, corals), arthropods (crustaceans, insects), cephalopods (octopuses, cuttlefish), fish and other vertebrates, Godfrey-Smith tells a captivating evolutionary story that is intended to close the gap between the mental and the physical, dispelling the illusion of a deep ontological chasm—helping us to see, in effect, how materialism could be true.

There are three philosophical ideas that lie at the heart of this project. One is a reorienting of the way we think about the infamous “hard problem” or “explanatory gap”. Godfrey-Smith wants to nudge us away from thinking of the problem in terms of qualia, qualities or the qualitative character of the mental. He criticises the idea that the paradigm case of a conscious experience involves staring at a Rothko painting and letting the colour wash over you. For a dualist, such cases have a special significance—they are the cases in which we get most intimately acquainted with a special type of property, distinct from any functional or structural property of the brain. But for a committed materialist, there are no such properties. We can get intimately acquainted with a painting, and perhaps form a special type of concept (a phenomenal concept) to think about our experience of the painting, but we are not encountering a special type of property.

If you think that qualities or qualia (conceived as a special type of property) are the source of the explanatory gap, but also think that there are no qualities or qualia, then you are naturally led to the view that there is no *serious* explanatory gap at all—just the illusion of one. This is Dennett/Frankish-brand illusionism (Dennett, 2017; Frankish, 2016). Taken to extremes, this is a view on which there are no conscious experiences in any animal, human or non-human. Godfrey-Smith asks us to stay off that path, and instead focus on a new set of paradigm cases that locate the explanatory gap in a different place.

These are cases in which the *subjective* character of conscious experience seems more prominent, and more gap-generating, than its qualitative character. Godfrey-Smith asks us to focus on experiences “where there is a certain kind of balance in place between a feeling of my own presence and a taking in of what is going on around me. … There is a scene, plus the feeling of being part of it” (p. 119). The new puzzle becomes that of explaining how, in a material world, this is possible. How did some animals evolve to be *subjects* with a point of view on the world—a world they feel themselves to be present in?

This new gap is more closeable (and more evolve-acrossable) than the old one. The question of consciousness is reconfigured as one about why there are *points of view*: action-guiding representations with a perspectival structure, in which a system constructs, and updates using sensory input, an inner model of itself as moving through, and interacting with, a stable external world. Godfrey-Smith rightly highlights Merker’s (2005, 2007) work on these inner models. As Merker emphasizes, at the core of any such model is the capacity to discriminate self from other in at least a basic sense—an animal needs to be able to distinguish exafferent (other-caused) changes to the stream of sensory input from reafferent (or self-caused) changes. Mechanisms of this type run very deep indeed in evolutionary history. As Barron and Klein (2016) have argued, it seems likely that insects have them.

So insects are conscious, then? Or have we been too willing to move the goalposts? I have a lingering sense that the problem of explaining Merker-style subjectivity is not really the “hard problem” any more, but something else—that we have not just reoriented ourselves, but set ourselves a different explanatory goal. One can clearly still ask the Chalmers question: why couldn’t perspectivally-structured egocentric modelling go on “in the dark”, free of any inner feel? Maybe to even ask such a question is to tacitly embrace dualism, but I’m not convinced of that.

The sense lingers with particular acuteness when I think about *Caenorhabditis elegans*, the famous 1 mm nematode worm with under 400 neurons (in contrast to our 100 billion). Nematodes have at least a simple version of Merker-style subjectivity. They exploit reafferent sensory input in the context of olfactory steering: they sweep their head from side to side, work out whether the odour gradient is getting stronger or weaker, and adjust their steering accordingly (Hendricks et al., 2012). The mechanism is beautifully simple, apparently relying on just a handful of neurons (Oullette et al., 2018). They register a difference between self and other in the most basic sense I can imagine. Intuitively, *that* could go on without subjective experience. Insects do a more complex kind of modelling, involving more sensory inputs, and this *might* mark an evolutionary watershed between non-conscious and conscious life, but it’s hard to see any intuitive reason why it would.

A different type of challenge to the Merker view comes not from nematodes but from humans. In humans, midbrain mechanisms (involving the superior colliculus and basal ganglia) seem to be sufficient for Merker-style subjectivity, as Merker himself has argued. They receive inputs from a variety of sources, and they orient us to our surroundings. Yet the content of conscious experience can differ from the content of these mechanisms. In cases of blindsight, people with damage to the primary visual cortex subjectively report having no conscious experience in a particular region of the visual field, yet still perform better than chance in forced-choice tasks that require access to information in that region (Ajina & Bridge, 2016). The information is reaching the midbrain but not the primary visual cortex—and conscious experience appears to come and go with the cortical processing. On the face of it, Merker has to side with a minority, notably including Phillips (2020), who think blindsight is not genuinely unconscious vision at all, but merely degraded conscious vision. The debate continues, but the pro-blindsight side remains the majority view, as far as I can tell, and undoubtedly has some strong cards (Michel & Lau, 2021).

Godfrey-Smith gestures towards the possibility of a different response to blindsight when he concedes that, in humans “a surprising amount can be done deep in the background” (p. 268) but suggests that processing which is pushed into the background in humans (and never registers in experience) might nonetheless register in experience in non-human animals. This is a fair speculation, but I am not sure what evidence supports it or could support it. The voice of LeDoux starts ringing in my ears: shouldn’t we assume that functions achieved unconsciously in humans are also achieved unconsciously in animals, unless there is some evidence against this? This is, after all, the approach we take with digestive functions, circulatory functions, and so on.

I think Godfrey-Smith himself feels the force of these challenges to the Merker view—which is why he brings in two further ideas. One is the idea of *global brain dynamic*s, especially synchronized neural oscillations, which have been implicated in both conscious perception and dreaming. Two things puzzle me about this. First, the suggestion of a link between these synchronized rhythms and conscious experience was very prominent in the early 1990s literature into which Chalmers first waded with the term “hard problem”. He would say: “the explanatory question remains: Why do the oscillations give rise to experience?” (Chalmers, 1995, p. 204). Godfrey-Smith thinks this question arises less easily for global patterns of activity than for more local patterns, but I don’t see why. Can’t we ask the Chalmers question just as easily either way? Whether the neural correlates of consciousness are local or global is a live empirical issue, but, for those who are convinced the hard problem is real, it will still be there whatever the answer.

Second, the move doesn’t help with the *C. elegans* problem, if it is a problem, since there are global brain dynamics involved in the regulation of behaviour (Kato et al., 2015) and sleep–wake cycles (Nichols et al., 2017) in *C. elegans*. This underlines the first point, since it makes the link between global brain dynamics and conscious experience feel particularly fragile and contingent. Maybe global brain dynamics in *C. elegans* indicate a conscious state when the animal is awake—but it is clearly possible that they don’t.

The third key idea in *Metazoa* is gradualism. Complex biological adaptations come into being gradually by tiny increments, with a borderline region in which the adaptation is neither clearly present nor clearly absent. The idea is that conscious experience is no exception—despite the strong temptation to make it an exception. Godfrey-Smith pushes against the widespread intuition that consciousness must be present or absent, on or off: that a particular state of a particular animal either determinately feels like something or does not. Anthony (2006) has called this the “intuition of sharpness” (see also Simon, 2017). I’ve written elsewhere about the absurdity of the suggestion that a cat neither  determinately does feel nor determinately does not feel (Birch, 2020a). Godfrey-Smith would say: it seems absurd because, for mammals, it is, but is it really so absurd for many invertebrates?

**Fig. 1:**
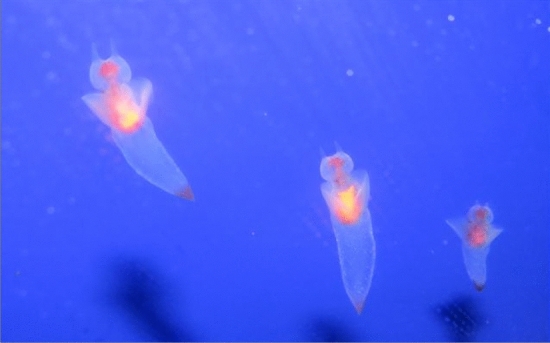
Sea angels. Rafael Guri / CC-BY-SA 3.0

This idea is intended to help when we think of nematodes, sea angels (a small sea slug, in case you were wondering; Fig. 1), other gastropods, insects, and so. It seems unbelievable, given materialism, that there could be some sharp threshold of entry to the conscious club—parents out, offspring in, a sudden hatching of consciousness into the world—as the complexity of sensorimotor integration and global brain dynamics gradually increases. Such a threshold would seem arbitrary and inexplicable. Some form of dualism would be needed to explain it. But, in Godfrey-Smith’s view, we don’t have to accept that there is any such threshold.

I do think a materialist probably has to embrace this deep form of gradualism. I can’t see any easy way to avoid it. At the same time, I can’t make sense of the claim that there are genuine borderline cases: that a nematode (or your preferred borderline case) neither determinately feels nor determinately does not feel. This is not allowed by my intuitive concept of conscious experience: for this to be true, that concept has to be revised. To the extent that deep gradualism is very difficult to understand or accept, this has to be added to the price of materialism.

What I particularly struggle with is the idea that, for some invertebrates, it may be that they neither determinately do nor determinately do not have moral status—and we neither determinately do nor determinately do not have moral obligations towards them. This will be an inevitable consequence of deep gradualism plus a tight relationship between consciousness and moral status (Birch, 2021; Cutter, 2017; Dunaway 2016). Can it be, for example, that we neither determinately do nor determinately do not have an obligation not to drop lobsters into boiling water? If you don’t see lobsters as a borderline case, just imagine a genuine borderline case. These cases put pressure on a materialist to develop a theory of degrees of moral status, somehow lining up with steps along the way from a determinately non-conscious state to a determinately conscious state. As Godfrey-Smith notes, “we will have to find new ways to think about those cases” (Godfrey-Smith, 2020, p. 274). The dualist’s ontology may be more complicated, but their moral world is simpler.

## The tip of the iceberg

For LeDoux, a neuroscientist best known for his work on emotion, conscious experience is the “tip of the mental iceberg”, associated with a very special type of information processing. Whereas Godfrey-Smith needs to push blindsight to the margins, LeDoux brings it to the centre of the picture. A lot can be done, he suspects, by unconscious processing alone. He stresses the need to use subliminal stimuli (e.g. backward masking) to separate conscious and unconscious processing of a stimulus—without that, he thinks, we can have no grip on what conscious processing really does.

Moreover, he insists on the primacy of verbal report for verifying that a stimulus was, or was not, consciously experienced. We can validate other indicators in humans by calibrating them against verbal report, but verbal report remains the gold standard. In these respects, he is probably closer than Godfrey-Smith to the mainstream in contemporary consciousness science.

When we ask what differs between subliminal and conscious perception, LeDoux thinks we are pushed by empirical evidence (see especially Lau & Rosenthal, 2011) in the direction of a Higher-Order Thought (HOT) view. He does not engage with longstanding criticisms of the HOT view, so readers (like me) who are not already convinced that the view is a promising one are unlikely to be persuaded. “HOT” has become something of an umbrella term for a family of different views, and it can be difficult to pin down what exactly the family has in common. If anything unites all HOT views, it is the idea that conscious experience involves a system representing its own mental states. There is disagreement among HOT proponents about the nature of the higher-order representation; the point of agreement is that there is one.

Any such view faces real trouble when we ask: what determines the content of a conscious experience—is it the first-order mental state or the higher-order thought? Suppose, for example, I perceive a blue sky but think that I am perceiving a red sky. What colour do I consciously experience: red or blue? If we say “blue”, the content of the HOT is now irrelevant to the content of the experience, raising the question of why it is needed at all. If we say “red”, the content of the HOT is now all-important, clashing with evidence that our experiences have fine-grained content that outruns our concepts (Neander 1998; Block, 2019; Carruthers 2019). As Block (2019, p. 202) puts it, “the cognitive system that according to [HOT] generates conscious experience is simply too coarse grained to explain normal human perceivers consciously seeing a million colors even though they have concepts of only a tiny fraction of those colors.” As far as I can tell, this well-known objection still stands.

Defenders of the HOT view have often been led, notoriously, to scepticism about consciousness in animals. Forming a HOT appears to require a sophisticated cognitive capacity—an ability to form conceptual representations of your own mental states—that many animals seem unlikely to possess. That traditional scepticism is certainly discernible in *The Deep History of Ourselves*. LeDoux emphasizes the links between higher-order thought, conscious experience and the frontal pole: a small region at the very front of the prefrontal cortex that is substantially enlarged in humans (in relation to the rest of the brain) in comparison with other great apes.

With this prefrontal-cortex-centred picture in the background, LeDoux goes on to draw a distinction between feelings and defensive survival circuits (or defensive survival states), with the former crucially depending on the cortex and the latter depending on subcortical regions such as the amygdala. It is a great mistake, he thinks, to develop drugs for psychiatric disorders that target only the subcortical mechanisms—we may think we are targeting fear or anxiety, but since these are cortex-dependent feelings, we are in fact targeting only the defensive survival circuits.

LeDoux is wary of explicit statements of scepticism about animal consciousness. Nonetheless, if your “default position is that behaviour is controlled nonconsciously until proven otherwise” (LeDoux, 2019, p. 328) and you also think that “the human brain is the only physical system that unequivocally possesses consciousness” (LeDoux, 2019, p. 328), the implication is that you think behaviour in non-human animals is controlled nonconsciously. Animals have survival circuits, but we have no reason to posit feelings in addition to those circuits. Elsewhere, however, he allows that “perhaps a nonverbal form of noetic awareness might exist in non-human primates, and possibly other mammals, and maybe even in birds” (p. 330). “Noetic awareness” is LeDoux’s term for the simplest form of conscious experience: experience which still involves higher-order representation, but without involving any kind of “reflective self-awareness” (p. 329).

This apparent tension can be resolved: LeDoux thinks a simple form of conscious experience *might* be present in other mammals and birds, but that there is no “demonstration” or “proof” of the type that would be provided by verbal reports of experience, and that a sceptical attitude is appropriate in the absence of such evidence. Note that even the “might” claim only extends to mammals and birds. Mammals have a neocortex, so the presence of prefrontal-cortical circuits supporting higher-order thoughts cannot be ruled out. Birds do not have a neocortex, but they have a dorsal pallium that is organizationally similar in important ways, despite lacking the laminated structure of the cortex (Güntürkün & Bugnyar, 2016). One might wonder: if you are prepared to speculate about birds on that basis, why not octopuses? But LeDoux does not want to go there. The neglect of invertebrates in the book is striking, especially in comparison with *Metazoa*.

All this makes “deep history” a slightly misleading name for LeDoux’s project in the early parts of the book. The first 260 pages sweep quickly^1^ across about 4 billion years of evolutionary history, from the dawn of life to the first humans; but this story, for LeDoux, is really one about the evolution of preconditions for, and precursors of, the human brain. For a HOT theorist, conscious experience as such has no deep history. There are discussions of the origin of life, the origin of sex, the origin of multicellularity, and so on, covering ground that will be reasonably familiar to readers well versed in the “major transitions” literature—but not because LeDoux thinks subjective experience entered the world at any of these points, but just because, since we are multicellular, sexually reproducing and alive, these transitions are part of “our story” in a broad sense. Whereas Godfrey-Smith structures his entire narrative around subjectivity, which he takes to have been present in a simple form hundreds of millions of years ago, subjectivity does not enter LeDoux’s story until chapter 12, when the focus shifts to modern humans.

## The search for better markers

**Fig. 2:**
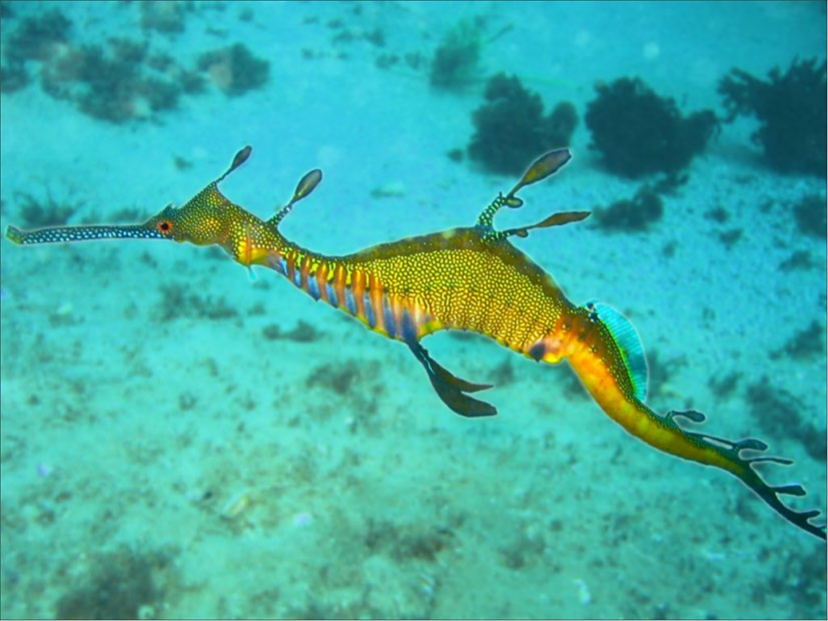
A common seadragon (*Phyllopteryx taeniolatus*). Richard Ling / CC-BY-SA 3.0

Which side am I on? I feel the pull of both books. On some points, I agree with LeDoux. A lot of information processing occurs unconsciously, and serious scientific inquiry into consciousness must somehow disentangle unconscious and conscious processing. We have some grip on how to do this for vision, in laboratory conditions, for humans and other primates, using techniques such as backward masking. We have no grip on how to do it for other animals, or outside the lab. Most animals, including seadragons (a seahorse-like Australian fish, with distinctive leaf-shaped skin lobes, in case you were wondering; Fig. 2) have never been studied at all in relation to consciousness. So I’m not *sure* that there is something it’s like to be a seadragon. I think we have enough evidence regarding fish to justify including them in the scope of animal welfare laws (Birch, 2017), but that does not mean the scientific question is settled. The word “sure” is not the right word here. We can’t say these questions are conclusively settled when they are not.

What I disagree with is LeDoux’s insistence on verbal report as a gold standard, as when he writes, for example, that “[t]he fact that animals can only respond nonverbally means there is no other response to help distinguish conscious from nonconscious processes” (LeDoux, 2019, p. 320) and disparages “nonverbal behavioral guesstimates” (LeDoux, 2019, p. 321). That is giving up much too easily! I share Shea and Bayne’s (2010) view that measuring consciousness with verbal report is akin to measuring the temperature of a liquid by putting your hand in it—it is our *initial* way of latching on to the phenomenon of interest, a starting point, not a measurement technique we can never transcend.

By using verbal report as a starting point, we can hope to find other cognitive and neural markers that are distinctive to conscious processing. If we find a substantial cluster of such markers, it would then seem dogmatic to insist that verbal report must always trump all the others—that a subject who is verbally reporting that they are not experiencing a stimulus should be believed unconditionally even when they display *all* of the other well-validated markers of experiencing it.

What are the “other cognitive and neural markers”? A science of animal consciousness would be easier to get off the ground if there were a consensus on this point, but there is not. We can point to some tentative but promising candidates for cognitive abilities that are facilitated by conscious perception (such as learning across temporal gaps, learning across sense modalities, and other elements of what Ginsburg and Jablonka have called “unlimited associative learning”; see Birch et al., 2020), and we can point to some possible neural markers (such as global ignition; see Mashour et al., 2020), but none is entirely uncontroversial. There is usually some debate about whether the marker is a genuine marker of conscious experience, or a marker of something causally upstream (e.g. “pre-consciousness”) or causally downstream (e.g. “reportability”) of conscious experience. So, if we want to study consciousness in non-mammals now, what should we look for?

What I think would be particularly valuable is to find a diverse cluster of plausibly consciousness-linked properties that *come and go together* across developmental and evolutionary time, such that, if an animal develops one, or if a species evolves one, it will tend to get the others—and if it loses one, it will tend to lose the others. Suppose, for example, that a seemingly disparate cluster of markers from our own case (e.g. global ignition, gamma oscillations, and learning across temporal gaps and across sense modalities) all turn out to be strongly correlated with each other, developmentally and evolutionarily, across the animal kingdom. This would suggest we had latched on to a property cluster “in the vicinity” of phenomenal consciousness, to use Nick Shea’s (2012) phrase. If we could also switch these markers on and off together by means of masking-like protocols (e.g. by manipulating the contrast or duration of a stimulus), that would be even more striking evidence (Birch, 2020b).

A debate would ensue about whether this “cluster in the vicinity” was in fact conscious experience itself, pre-conscious processing, or post-conscious processing, and I don’t want to understate the difficulty of resolving that debate (see Phillips, 2018 on Shea). Moreover, we would still be left with the hard problem: the problem of explaining why this property cluster, and not something else entirely, is linked to there being something it’s like to be you. But we would have made progress. Animals found to possess the cluster could be regarded as empirically well-supported candidates for consciousness, pending further evidence from the human case. Shea and Bayne (2010) advocate essentially this strategy for identifying the minimally conscious state in humans with severe brain damage (albeit with more emphasis on neural markers and less on cognitive markers)—and, in my view, the programme of identifying consciousness in these cases should be closely allied with the search for consciousness in animals. In both contexts, we should be willing to treat an “empirically well-supported candidate” on the assumption that it *is* conscious when thinking about the ethics of potentially painful procedures.

If this research programme were to be pursued for a sustained period, my hunch is that it would end up vindicating a Godfrey-Smith-esque picture of the natural world, on which at least some invertebrates—the coleoid cephalopod molluscs and some arthropods—would be widely regarded as conscious beings. It might or might not also vindicate Godfrey-Smith's gradualism, since I see a property-cluster based methodology as neutral on the issue of borderline cases: it allows for the possibility of there being borderline cases of the cluster in question, but also leaves open the possibility of a sharp boundary. So I suspect that Godfrey-Smith may well be right about the distribution of consciousness in nature, and could be right about gradualism too, and yet I also think LeDoux is right to demand higher quality evidence before regarding the matter as *conclusively* settled. The insistence on the need to distinguish unconscious and conscious processing is one the emerging field of animal consciousness research can and should meet.

## Footnotes

1 Sometimes too quickly—as when LeDoux writes that “In placental mammals the fetus remains in the placenta (a modification of the amniotic membranes in reptiles). When the placenta breaks, releasing fluid, it is a sign that birth is near” (LeDoux 2019, p. 170). This is incorrect.
